# Dapagliflozin reverses the imbalance of T helper 17 and T regulatory cells by inhibiting SGK1 in a mouse model of diabetic kidney disease

**DOI:** 10.1002/2211-5463.13147

**Published:** 2021-05-01

**Authors:** Dan Wang, Zikun Zhang, Zekun Si, Yanlin Yang, Shuangshuang Li, Yaoming Xue

**Affiliations:** ^1^ Department of Endocrinology and Metabolism Nanfang Hospital Southern Medical University Guangzhou China

**Keywords:** diabetic kidney disease, inflammatory response, serum, glucocorticoid‐regulated kinase 1, sodium, glucose co‐transporter 2, Th17/Treg cell imbalance

## Abstract

An imbalance between T helper 17 (Th17) and T regulatory (Treg) cell subsets contributes to the pathogenesis of diabetic kidney disease (DKD). However, the underlying regulatory mechanisms that cause this imbalance are unknown. Serum/glucocorticoid‐regulated kinase 1 (SGK1) has been suggested to affect Th17 polarization in a salt‐dependent manner, and sodium/glucose cotransporter 2 inhibitors (SGLT2i) have been demonstrated to regulate sodium‐mediated transportation in the renal tubules. This study aimed to evaluate the potential benefits of dapagliflozin (Dap) on DKD, as well as its influence on shifting renal T‐cell polarization and related cytokine secretion. We treated male db/db mice with Dap or voglibose (Vog) and measured blood and kidney levels of Th17 and Treg cells using flow cytometry. We found that Th17 cells were significantly increased, while Treg cells were significantly decreased in diabetic mice. Moreover, Dap suppressed the polarization of Th17/Treg cells by inhibiting SGK1 in diabetic kidneys, and this was accompanied by attenuation of albuminuria and tubulointerstitial fibrosis independent of glycemic control. Taken together, these results demonstrate that the imbalance of Th17/Treg cells plays an important role in the progression of DKD. Moreover, Dap protects against DKD by inhibiting SGK1 and reversing the T‐cell imbalance.

## Graphical abstract

An imbalance between Th17 and Treg cell subsets contributes to the pathogenesis of diabetic kidney disease. We found that Th17 cells were increased, while Treg cells were decreased in diabetic mice. Dapagliflozin suppressed the polarization of Th17/Treg cells by inhibiting SGK1 in diabetic kidneys, and this was accompanied by attenuation of albuminuria and tubulointerstitial fibrosis independent of glycemic control.

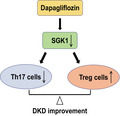

**Abbreviations**
Dap: dapagliflozin
DKD: diabetic kidney disease
DM: diabetes mellitus
ESRD: end‐stage renal disease
IL: interleukin
PMA: phorbol 12‐myristate 13‐acetate
SGK1: serum/glucocorticoid‐regulated kinase 1
SGLT2i: sodium/glucose cotransporter 2 inhibitors
TGF‐β: tumor growth factor‐β
Th17: T helper 17
TNF‐α: tumor necrosis factor‐α
Treg: T regulatory
Vog: voglibose

Diabetic kidney disease (DKD), characterized by progressive albuminuria and reduced glomerular filtration, has become the leading cause of end‐stage renal disease (ESRD) worldwide [1]. With the prevalence of diabetes mellitus (DM) doubling in recent decades, DKD has accounted for about half of all ESRD etiologies [2]. The pathogenesis of DKD is multifactorial, and one identified pathogenic factor is an imbalance of T helper 17 (Th17) and T regulatory (Treg) cells. Early studies confirmed the accumulation of activated T cells and inflammatory factors in kidneys of DKD patients [3]. Further studies indicated that the accumulation of T cells in kidneys of DKD patients was characterized by an upregulation of pro‐inflammatory T cells (Th17 and Th1) and pro‐inflammatory cytokines [interleukin (IL)‐17, tumor necrosis factor‐α (TNF‐α), and IL‐6], and downregulated anti‐inflammatory T cells (Treg cells) and anti‐inflammatory cytokines [(IL‐10 and tumor growth factor‐β (TGF‐β)] [4, 5, 6, 7]. Serum/glucocorticoid‐regulated kinase 1 (SGK1) is a serine/threonine kinase that is constitutively expressed in various tissues [8]. Despite its regulatory efficacy on cellular Na+ transport [9], SGK1 can influence the development of Th17 cells via increasing cellular salinity. It has been confirmed that high salinity can induce SGK1 expression, while silencing SGK1 significantly can reduce the production of Th17 cells induced by high salt [10]. The underlying mechanism may be related to the activation of the SGK1/phosphorylated (p‐)Foxo1/IL‐23R pathway, and the stabilization and enhancement of Th17 cells [11]. However, whether SGK1 promotes the progression of DKD via stimulating an imbalance of Th17 and Treg cells remains unknown.

SGK1 is activated by excessive circulating glucose, which subsequently stimulates a number of ion channels, such as renal sodium/glucose cotransporter 2 (SGLT2) and epithelial sodium channel [12]. Sodium/glucose co‐transporters (SGLTs) belong to the solute carrier 5 family of glucose active transporters, which mainly exert the physiological transport of glucose via SGLT1 and SGLT2 subtypes [13, 14]. It has been found that the high‐carrying capacity, low‐affinity SGLT2 transporter is located almost exclusively in renal proximal tubular epithelial cells and is responsible for ~ 97% of the reabsorption of filtered glucose [15]; the remaining 3% is reabsorbed by SGLT1 under otherwise normal physiological conditions [16]. Glucose kinetic studies have demonstrated that SGLT2 inhibitors decrease the maximal carrying capacity of tubular glucose reabsorption, thereby increasing urinary glucose excretion and decreasing plasma glucose concentrations [17]. Clinically, SGLT2 inhibitors, a novel type of hypoglycemic agents, have been shown to confer antihypertensive, weight loss, uric acid lowering, cardioprotective, and renal protection properties, in addition to their hypoglycemic characteristics [18, 19, 20, 21, 22, 23]. Interestingly, a previous study showed that treatment with the SGLT2 inhibitor empagliflozin improved glycemic control and cardiac diastolic function and reduced expression of cardiac SGK1 in diabetic mice [12]. Based on the above findings, we aimed to evaluate the potential benefits of the SGLT2 inhibitor dapagliflozin (Dap) on DKD in diabetic mice, focusing on its influence on reversing the SGK1‐mediated Th17/Treg cell imbalance.

## Materials and methods

### Animals

Diabetic db/db mice and C57BLKS/J mice (8 weeks old) were purchased from Nanjing Institute of Biomedicine affiliated with Nanjing University (Nanjing, China). Animals were housed in a specific pathogen‐free room at 20–25 °C and were maintained on a continuous 12:12‐h light–dark cycle with free access to water and standard chow. After 2 weeks of adaptive housing, mice were randomly allocated into groups. Diabetic db/db mice were randomized to receive either normal saline as a vehicle control, Dap (1 mg·kg^−1^, Meilun, China, MB1917), or voglibose (Vog; 0.6 mg·kg^−1^, Meilun, MB2024) (*n* = 6 each group) [24]. The α‐glucosidase inhibitor Vog, used as a positive control, is also widely used to improve postprandial hyperglycemia without proven renoprotective effects [25]. Non‐diabetic C57BLKS/J mice that received normal saline served as controls (*n* = 6). All treatments and vehicle were administered to animals daily by gavage for 12 consecutive weeks. Body weights of all mice were measured once a week, blood glucose was measured every 4 weeks, and urine volumes were measured at 16 and 22 weeks after treatment. Blood was collected from the tail vein for blood glucose measurements. On the last day of week 22, animals were sacrificed, and renal tissues and blood were collected for further analyses. All experimental protocols were approved by the Ethics Committee of the Southern Medical University (No: NFYY‐2017‐39), and all animal studies were performed in accordance with the Institutional Animal Care Guidelines.

### Immunohistochemistry

Mice were perfused with cold PBS, and kidney tissue samples were collected, fixed in 4% paraformaldehyde, embedded in paraffin, and sectioned into 3‐μm slices using a microtome (Leica RM 2235, Wetzlar, Germany). The tissues were then mounted onto glass slides and stained with PAS (Loogene, Beijing, China) and Masson chrome stain (Maiwei, Xiamen, China) as previously described [26]. For immunohistochemical analysis, the slides were stained with an anti‐SGK1 polyclonal antibody (1 : 1000; Abcam, Cambridge, UK, ab43606) or an anti‐SGLT2 polyclonal antibody (1 : 200; Proteintech, Chicago, IL, USA, 24654‐1‐AP), followed by an HRP‐conjugated secondary antibody (1 : 2000), as described previously [27]. All images were obtained using an Olympus microscope (Olympus, Tokyo, Japan).

### Biochemical analysis

After a 5‐day acclimation period, mice were housed in metabolic chambers with free access to drinking water and food for 24 h to collect urine samples at the indicated durations. After centrifugation for 10 min at 3000 ***g***, urinary albumin and creatinine levels were assayed using an ELISA Kit (Franke, Shanghai, China). The blood samples were placed into EP tubes for anticoagulation at 4 °C and then centrifuged for 10 min at 3000 ***g***. Serum IL‐10 (Catalog Number: E0056m) and IL‐17 (Catalog Number: E0063m) concentrations were measured using an ELISA Kit (EIAab, Wuhan, China). The assays were performed according to the manufacturer's instructions, and absorbance was measured using the Bio‐Rad iMark Microplate Reader.

### Flow cytometry analyses

Single‐cell suspensions were obtained following centrifugation of blood and kidney samples. In brief, 200 μL of whole blood was collected and incubated with 2 mL of erythrocyte lysates at 37 °C for 2–3 min. The samples were centrifuged at 250 ***g*** for 5 min and washed twice with PBS. Kidney tissues were cut into pieces and digested with 0.25% trypsin and 0.01% collagenase type II at 37 °C for 20 min. After centrifugation at 250 ***g*** for 5 min, solutions were filtered through a 200‐mesh sieve. The filtrates were centrifuged at 94 ***g***. for 5 min and were incubated with 2 mL of erythrocyte lysates at 37 °C for 2–3 min. The samples were also centrifuged and washed for further use.

For analysis of intracellular cytokine production, kidney and blood mononuclear cells were stimulated with 150 ng·mL^−1^ phorbol 12‐myristate 13‐acetate (PMA) and 1 μg·mL^−1^ ionomycin in the presence of GolgiStop (BD Biosciences, San Diego, CA, USA) for 6 h. For Th17 cell analysis, the samples were digested and fixed with 100 μL of intracellular fixation and permeabilization buffer (eBioscience Catalog No. 88‐8824‐00, San Diego, CA, USA) for 20 min at room temperature in the dark and then washed with 2 mL permeabilization buffer twice. After centrifugation, the samples were resuspended in 100 µL permeabilization buffer and stained with a PE‐labeled anti‐mouse CD4 antibody (Catalog No. 12‐0041‐82; Invitrogen, Carlsbad, CA, USA) and a FITC‐labeled anti‐mouse IL‐17A antibody (Catalog No. A15377; Invitrogen) for 30 min in the dark before they were washed with 2 mL of permeabilization buffer. After addition of 500 μL staining buffer (eBioscience Catalog No. 00‐4222‐26), the cells were analyzed using the FACSCalibur cytometer (Becton Dickinson, New York, NY, USA). For analysis of Treg cells, the samples were digested before fixation and permeabilization, and were then stained with a PE‐labeled anti‐mouse CD4 antibody (Catalog No. 12‐0041‐82; Invitrogen) for 30 min at 4 °C. The samples were incubated with Foxp3 transcription factor staining buffer (eBioscience Catalog No. 00‐5523) for 30 min at 4 °C and a FITC‐labeled anti‐mouse FoxP3 antibody (Catalog No. A18662; Invitrogen) for 30 min in the dark. The CD4^+^Foxp3^+^ cells were considered Treg cells. The scatter patterns of lymphocytes are shown in Figs S1–S4.

### Magnetic bead cell sorting

Preparation of single‐cell suspension from kidney tissue: Minced kidney tissues were digested with 0.25% trypsin and 0.01% type II collagenase for 20 min at 37 °C, and enriched by centrifugation at 250 ***g*** for 5 min. The cell pellets were resuspended with RPMI 1640 media (C22400500BT; Gibco, Grand Island, NE, USA) containing 10% FCS (10091148; Gibco), 1 mm sodium pyruvate (S104174; Aladdin, Shanghai, China), 50 μm 2‐mercaptoethanol (BB‐92003; BestBio, Beijing, China), 2 mm glutamine, 25 mm HEPES, and 1 × nonessential amino acid (1140050; Gibco), and were passed through 200‐mesh screen. A total of 1 × 10^8^ cells was used for cell sorting. Anti‐mouse CD4 and CD62 MicroBeads were used to select CD4+ CD62L+ naive T cells, as recommended by the manufacturer (Miltenyi Biotec, Bergisch Gladbach, Germany; 130‐106‐643). The cells were gated on lymphocytes based on FSC‐SSC. The Th17 cells were collected from CD4+ CD62L+ cells after stimulation with recombinant TGF‐β (3 ng·mL^−1^; PeproTech, Rocky Hill, CT, USA), IL‐6 (300 IU·mL^−1^; PeproTech), and IL‐23 (300 IU·mL^−1^; PeproTech) for 90–108 min, at a cell density of 1 × 10^6^ cells per mL. Approximately 5000 CD4+ T cells were acquired using cellquest software (Becton Dickinson, New York, NY, USA), and the mean fluorescence intensities of CD4 were determined using flow cytometry. The cells were gated on FSC‐SSC plots and were further plotted against CD4 and IL‐17. The CD4+ IL‐17+ cells were identified as Th17 cells. The Th17 cells were used for subsequent flow cytometry and western blot analyses.

### Western blot analysis

Equal amounts of protein (20 μg) extracted from Th17 cells using RIPA (Biyuntian, Shanghai, China) were electrophoresed using 10% SDS/PAGE, transferred to Polyvinylidene fluoride membranes, blocked, and then incubated at 4 °C overnight with the following primary antibodies: SGK1 (1 : 500; Abcam, ab43606), p‐Foxo1 (1 : 500; Abclonal, Boston, MA, USA, AP0176), and IL‐23R (1 : 500; Abcam, ab175072). The blots were then incubated with an HRP‐conjugated secondary antibody (1 : 2500; Proteintech) at room temperature for 1 h and visualized using a Bio‐Rad Gel Doc EZ Imaging System (Bio‐Rad, Hercules, CA, USA) with enhanced chemiluminescence reagents (Millipore, Boston, MA, USA). All western blot analyses were performed at least three times, and the protein band densities were quantified using imagej software (NIH, National Institutes of Health, Bethesda, MA, USA).

### Statistical analysis

Continuous variables are presented as mean ± standard deviation (*x* ± SD). prism 5 (GraphPad software Inc., San Diego, CA, USA) and spss22.0 statistical software (IBM, International Business Machines Corporation, Armonk, USA) were used for statistical analysis. One‐way ANOVA with SNK test (each group has equal variance) or Dunnett's T3 test (equal variances not assumed) was used to assess statistical significance among the four groups. Comparisons of body weight and random blood glucose between groups were performed using repeated measurements (RM) followed by the SNK test or Dunnett's T3 test. A *P*‐value < 0.05 was considered statistically significant.

## Results

### Dapagliflozin reduces urinary albumin in DM mice without significantly affecting blood glucose and body weight

**Fig. 1:**
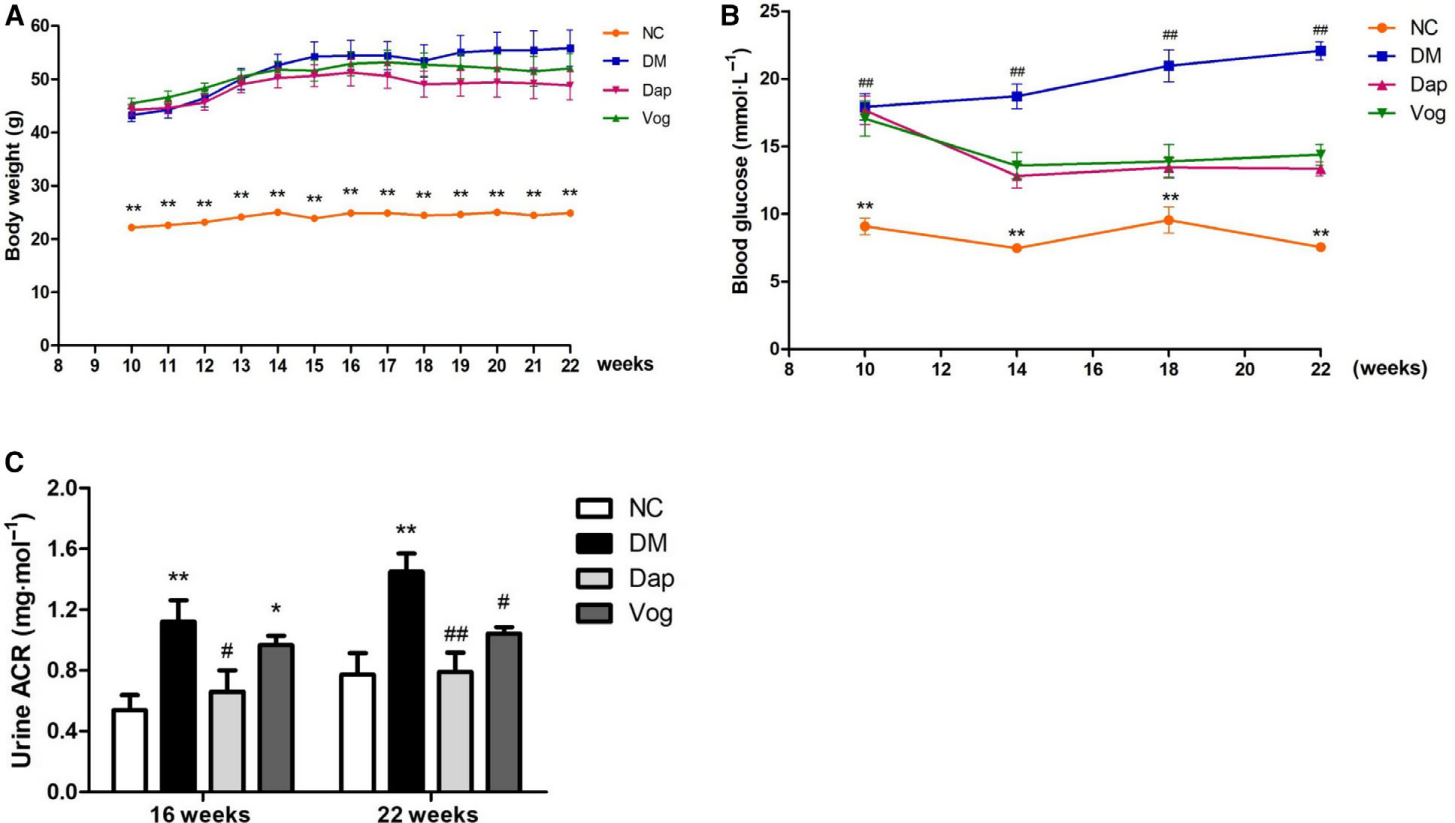
Dap treatment partly reversed random blood glucose and urinary albumin/creatinine ratio, but not body weight, in db/db mice. Mice were randomly allocated into the following groups: nondiabetic C57BLKS/J mice (*n* = 6); diabetic db/db mice treated with normal saline, Dap (1 mg·kg^−1^), or voglibose (Vog; 0.6 mg·kg^−1^) (*n* = 6 each group). (A) Body weight, (B) random blood glucose, (C) urinary albumin/creatinine ratio. Quantitative data are presented as mean ± SD. One‐way ANOVA with SNK test or Dunnett's T3 test was used for statistical analysis. *, *P* < 0.05 compared with the NC group, **, *P* < 0.01 compared with the NC group, #, *P* < 0.05 compared with the DM group, and ##, *P* < 0.01 compared with the DM group.

Body weight and random blood glucose were significantly higher in the DM, Dap, and Vog groups compared with the NC group. Body weight was not significantly different among mice in the Dap, Vog, and DM groups. Blood glucose was significantly lower in the Dap and Vog groups compared with the DM group (Fig. 1A,B). The ratio of urinary albumin/creatinine in the DM group was significantly higher compared with the NC group, but was significantly lower in the Dap group compared with the Vog and DM groups (Fig. 1C).

### Dapagliflozin improves renal pathological changes and inhibits SGLT2 and SGK1 expression

**Fig. 2:**
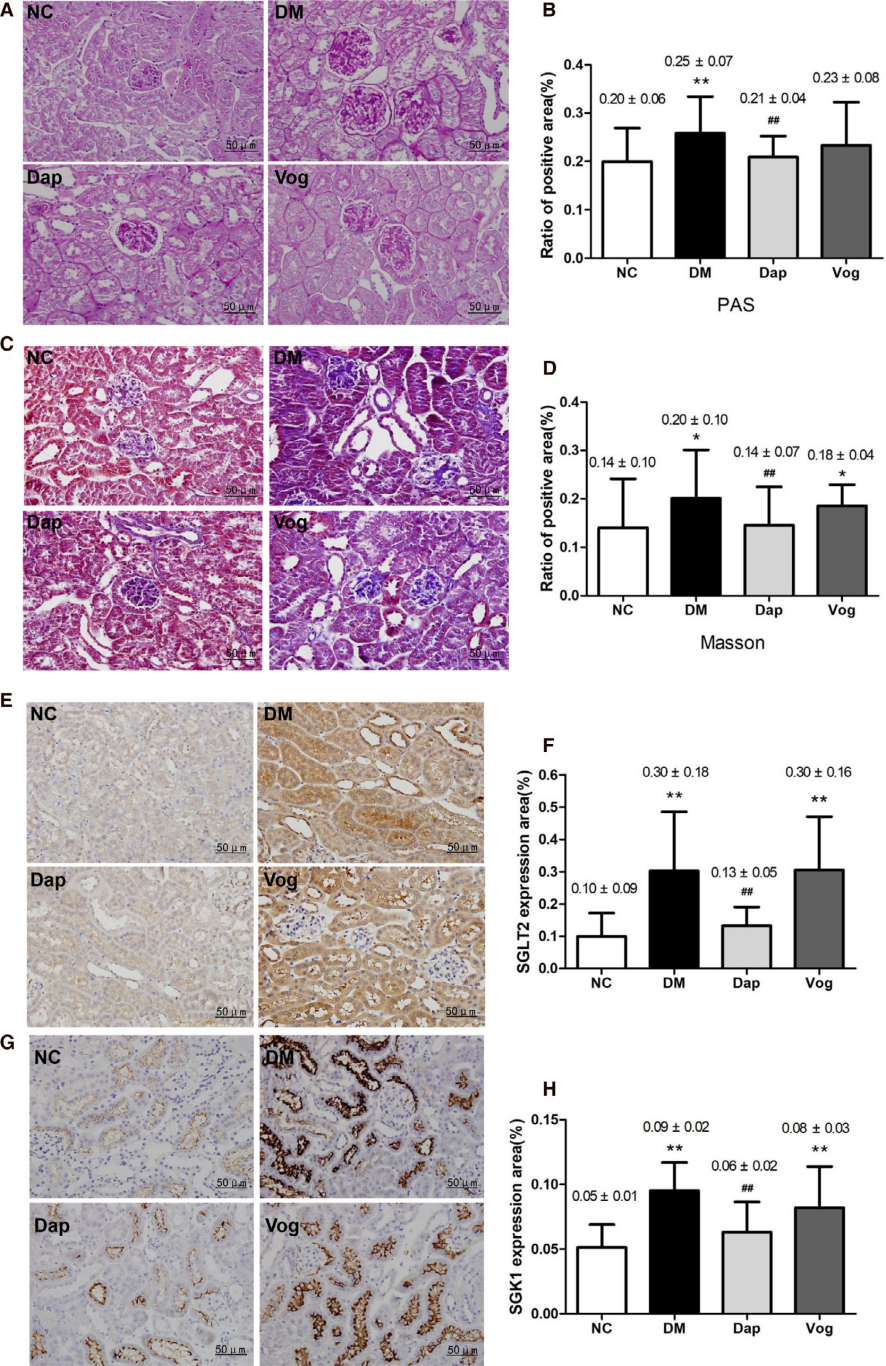
Dap treatment partly reversed renal fibrosis and inhibited SGLT2 and SGK1 expression in db/db mice. We did not observe any significant difference in renal fibrosis between the DM and Vog groups Mice were randomly allocated into the following groups: nondiabetic C57BLKS/J mice (*n* = 6); diabetic db/db mice treated with normal saline, Dap (1 mg·kg^−1^), or voglibose (Vog; 0.6 mg·kg^−1^) (*n* = 6 each group). Kidney tissue samples were fixed in 4% paraformaldehyde and stained with PAS and Masson. For immunohistochemical analysis, the slides were stained with anti‐SGK1 or anti‐SGLT2 polyclonal antibodies, followed by HRP‐conjugated secondary antibody. (A) Representative images of PAS staining and (B) semi‐quantitative analysis; (C) representative images of Masson staining and (D) semi‐quantitative analysis; (E) representative images of immunohistochemical staining for renal SGLT2 and (F) semi‐quantitative analysis; (G) representative images of immunohistochemical staining for renal SGK1 and (H) semi‐quantitative analysis. Error bar = 50 μm. Quantitative data are presented as mean ± SD. One‐way ANOVA with SNK test or Dunnett's T3 test was used for statistical analysis. *, *P* < 0.05 compared with the NC group, **, *P* < 0.01 compared with the NC group, #, *P* < 0.05 compared with the DM group, and ##, *P* < 0.01 compared with the DM group.

PAS and Masson staining showed that kidney fibrosis was increased in the DM group, as evidenced by a thickened glomerular basement membrane, widened mesangial matrix, and an obvious fibrotic renal tubulointerstitium. Treatment with Dap alleviated the renal fibrosis, as evidenced by attenuated thickness of the renal basement membrane and the width of mesangial matrix. We did not observe any significant difference in renal fibrosis between the DM and Vog groups (Fig. 2A‐D). Furthermore, immunohistochemical analyses showed that SGLT2 protein expression and SGK1 protein expression were significantly higher in the kidneys of the DM group compared with the NC group, but significantly lower in the Dap group compared with the DM group. In addition, SGLT2 expression was not significantly different between the Vog and DM groups, while SGK1 expression was reduced in the Vog group compared with the DM group (Fig. 2E–H).

### Dapagliflozin improves the Th17/Treg cell imbalance in peripheral blood and renal tissues of DM mice

**Fig. 3:**
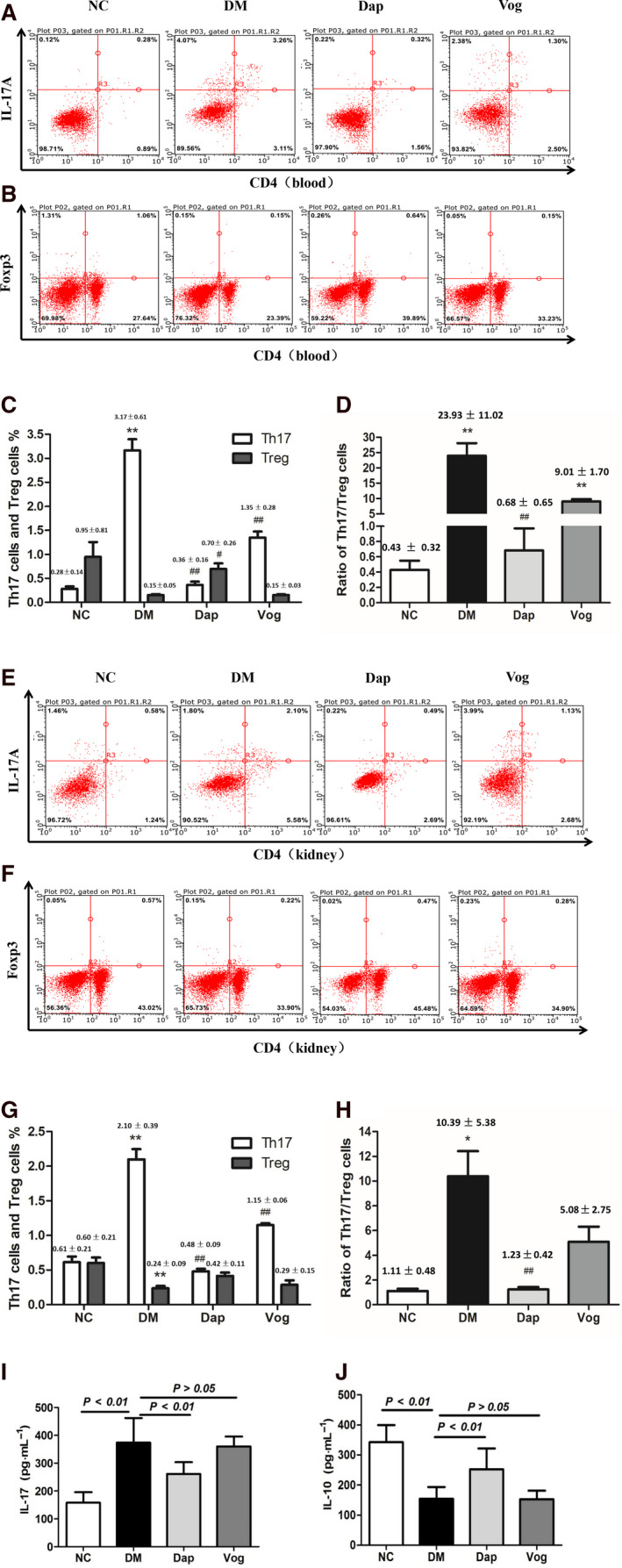
Dap treatment changed the subpopulations of Th17 and Treg cells in db/db mice (*n* = 6) after 12 weeks of intervention. Single‐cell suspensions were obtained following centrifugation of kidney and blood samples, followed by stimulation with 150 ng·mL^−1^ PMA and 1 μg·mL^−1^ ionomycin in the presence of GolgiStop for 6 h. For Th17 cell analysis, the samples were stained with a PE‐labeled anti‐mouse CD4 antibody and then were incubated with a FITC‐labeled anti‐mouse IL‐17A antibody. For analysis of Treg cells, the antibodies used included a PE‐labeled anti‐mouse CD4 antibody and a FITC‐labeled anti‐mouse FoxP3 antibody. (A) Flow cytometry measurements for blood Th17 cells; (B) flow cytometry measurements for blood Treg cells; (C) quantitative analyses for blood Th17 and Treg cells; (D) quantitative analyses for the blood Th17/Treg cell ratio; (E) flow cytometry measurements for kidney Th17 cells; (F) flow cytometry measurements for kidney Treg cells; (G) quantitative analyses for kidney Th17 and Treg cells; (H) quantitative analyses for the kidney Th17/Treg cell ratio; (I) serum levels of IL‐17 in each group; and (J) serum levels of IL‐10 in each group. Quantitative data are presented as mean ± SD. One‐way ANOVA with SNK test or Dunnett's T3 test was used for statistical analysis. *, *P* < 0.05 compared with the NC group, **, *P* < 0.01 compared with the NC group, #, *P* < 0.05 compared with the DM group, and ##, *P* < 0.01 compared with the DM group.

Compared to the NC group, the number of Th17 cells was significantly increased in both blood and kidneys from the DM group, while the number of Treg cells was decreased and the ratio of Th17/Treg cells was significantly increased. Further analysis revealed that the increased serum IL‐17 and decreased serum IL‐10 levels were positively correlated with DM status, which we did not observe in mice from the NC group. Treatment with Dap reversed the changes in the serum inflammatory cytokines and the imbalance of Th17/Treg cells, while treatment with Vog did not significantly affect the serum inflammatory cytokines and T‐cell subsets (Fig. 3A–J).

### Dapagliflozin inhibits SGK1, p‐Foxo1, and IL‐23R expression in Th17 cells from DM kidneys

**Fig. 4:**
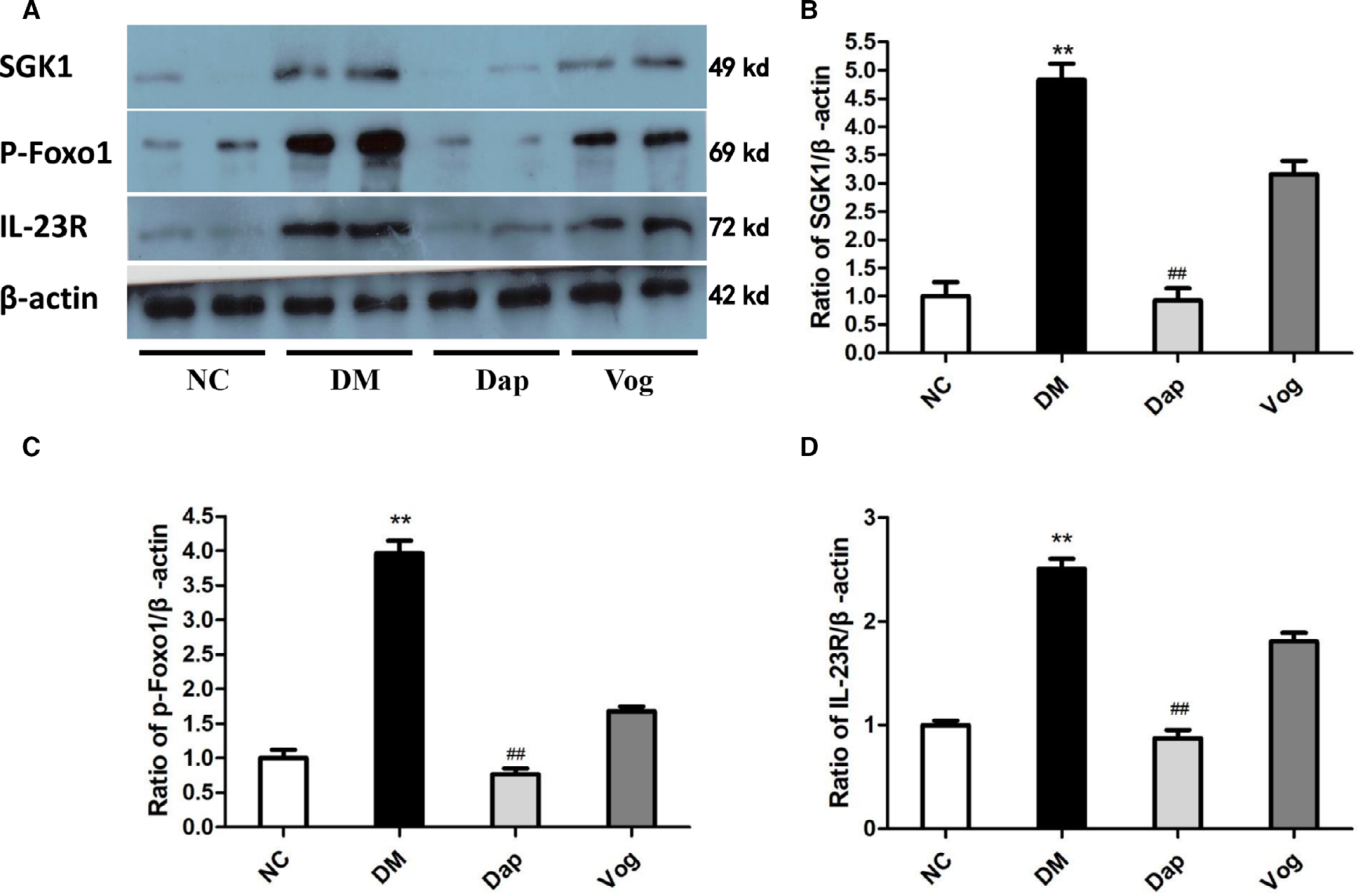
Dap treatment reduced the protein levels of SGK1, p‐Foxo1, and IL‐23R in kidney Th17 cells of db/db mice. Mice were randomly allocated into the following groups: Th17 cells were isolated from the kidneys in nondiabetic C57BLKS/J mice (NC), and diabetic db/db mice treated with normal saline (DM), Dap (1 mg·kg^−1^), or voglibose (Vog; 0.6 mg·kg^−1^) (*n* = 6 for each group). The Th17 cells were collected from CD4+ CD62L+ naive T cells after stimulation with recombinant TGF‐β (3 ng·mL^−1^), IL‐6 (300 IU·mL^−1^), and IL‐23 (300 IU·mL^−1^). Total cell proteins were extracted from Th17 cells for western blot analyses. Equal amounts of protein (20 μg) extracted from Th17 cells were incubated with the following primary antibodies: SGK1, p‐Foxo1, and IL‐23R. (A) representative images for SGK1, p‐Foxo1, and IL‐23R expression; (B) semi‐quantitative analysis for SGK1 expression; (C) semi‐quantitative analysis for p‐Foxo1 expression; (D) semi‐quantitative analysis for IL‐23R expression. Quantitative data are presented as mean ± SD. One‐way ANOVA with SNK test or Dunnett's T3 test was used for statistical analysis. **, *P* < 0.01 compared with the NC group; ##, *P* < 0.01 compared with the DM group.

To further clarify the effect of SGK1 on Th17 cell differentiation, Th17 cells were isolated from renal tissues and measured using western blot analysis. The results showed that the levels of SGK1 pathway‐related proteins, including p‐Foxo1 and IL‐23R, were significantly increased in the DM group. Treatment with Dap inhibited SGK1, p‐Foxo1, and IL‐23R protein expression in the kidneys from the DM mice, while treatment with Vog did not significantly affect the expression of these proteins (Fig. 4A–D).

## Discussion

In China, about 20–40% of DM patients have DKD [28], which has been attributed to metabolic–hemodynamic changes, oxidative stress, inflammatory responses, and the renin–angiotensin system [29]. The current study evaluated the potential benefits of the SGLT2 inhibitor Dap on DKD in a diabetic mouse model, focusing primarily on the ability of Dap to influence the Th17/Treg cell imbalance.

We confirmed that db/db mice gradually develop DKD, as evidenced by our histological analyses, as well as by increases in body weight, blood glucose levels, and the ratio of urinary protein/creatinine during DM progression. Increasing evidence indicates that T‐cell‐induced inflammatory responses play a vital role in the pathogenesis of DKD in DM [30] because T cells accumulate in the renal juxtaglomerular apparatus [3]. Furthermore, an imbalance of pro‐inflammatory T cells (Th17 and Th1) and anti‐inflammatory T cells (Treg cells) [4] was reported in a previous study. Results of our study confirmed these findings, showing that the Th17 cells in the blood and kidneys were significantly increased, while Treg cells were significantly decreased in diabetic mice. We also found increases in serum IL‐17 and decreases in serum IL‐10 levels. These results suggest that an imbalance of T cells and related changes in inflammatory factors are important determinants of DKD pathogenesis.

Th17 cells are a subset of CD4+ helper T cells, which are widely believed to induce inflammation in the pathogenesis of autoimmune diseases by producing cytokines such as IL‐17, TNF‐α, and IL‐6. Th17 cells counteract Treg cells, which function by maintaining immune homeostasis and tolerance by secreting anti‐inflammatory cytokines IL‐10 and TGF‐β [31]. Our findings are consistent with previous clinical studies that showed that the Th17/Treg cell ratio was significantly higher in type 2 DM (T2DM) patients with DKD compared to T2DM patients without DKD or healthy controls [5]. Another *in vivo* study showed that the mycophenolate mofetil improved pathological kidney changes in DM patients, which were accompanied by reduced numbers of Th17 cells in the kidney [6]. Taken together, these findings confirm the previous hypothesis that a Th17/Treg cell imbalance plays a key role in the development and progression of DKD [7].

We also found that circulating IL‐17 levels were higher, IL‐10 levels were lower, and there was an imbalance of Th17/Treg cells in DM mice compared with controls. These findings suggest that a Th17/Treg cell imbalance accelerates the development of DKD via inducing inflammatory responses. Previous studies indicated that SGK1 regulates the Th17/Treg cell balance via influencing cell salinity [10]. Indeed, Wu *et al*. [11] confirmed that high‐salt treatment induced SGK1 expression, and exposure of IL‐23R‐deficient T cells to IL‐23 reduced SGK1 expression. In contrast, SGK1 deficiency does not prevent Na+‐mediated Th17 differentiation in an IL‐23R‐dependent manner [11]. Furthermore, IL‐23R and IL‐17A expression in CD4+ memory T cells lacking Foxo1 increased compared with wild‐type cells. IL‐23R could also be trans‐activated by the RORγt promoter, an important IL‐17 transcription regulator, which can be inhibited by Foxo1. Wu *et al*. [11] also indicated that activation of SGK1 can sequentially inactivate Foxo1 via phosphorylation, subsequently leading to the activation of RORγt by relieving the inhibition of RORγt, and promoting IL‐23R expression, ultimately resulting in Th17 differentiation [11]. Results of our study indicate that the activation of the SGK1, p‐Foxo1, and IL‐23R pathways is associated with a Th17/Treg imbalance, suggesting that SGK1 regulates the pathogenesis of DKD via influencing the Th17/Treg cell ratio. Given that there are few reports on the influence of SGK1 on other immune cells in the kidney, the influence of Dap on various immune cells during DKD development warrants further research.

SGK1 can stimulate SGLT2 activation, and Dap has been recognized as a hypoglycemic agent that inhibits SGLT2 in renal tubular epithelial cells [32]. Interestingly, SGLT2 resorbs Na+ in addition to glucose in urine, potentially increasing salinity, which may induce the imbalance of Th17/Treg cells and lead to DKD pathogenesis. Accordingly, Dap may confer renal protection by inhibiting SGLT2‐mediated high salinity and the imbalance of Th17/Treg cells in DM mice. This was confirmed by our findings that Dap treatment decreased renal SGK1 protein expression, reduced circulating IL‐17 levels, increased circulating IL‐10 levels, and significantly attenuated the pathological kidney changes in DM mice, all of which were not observed in mice treated with Vog. Vog reduces blood glucose levels by inhibiting intestinal sugar absorption, which differs from the glucose‐lowering mechanism of Dap. Thus, Dap may reverse the imbalance of Th17/Treg cells by inhibiting Na+ resorption.

These results indicate that Dap confers protection in DM patients, primarily via its favorable influence on Th17/Treg cells and inflammation, rather than via its hypoglycemic effects. These mechanisms may be important for understanding the renal protective efficacy of Dap in DM patients.

In conclusion, we found that increased expression of SGLT2 could result in an imbalance of Th17/Treg cells in the kidney via activation of the SGK1/p‐Foxo1/IL‐23R pathway and that this underlies DKD pathogenesis. Accordingly, Dap treatment may confer renal protection in DM patients via reversing the SGK1‐mediated Th17/Treg cell imbalance.

## Conflict of interest

The authors declare no conflict of interest.

## Author contributions

DW designed the study, performed the experiments, and prepared the manuscript. ZZ and ZS performed the experiments and prepared the manuscript, researched data, and assisted with revising of the manuscript. YY and SL proposed the hypothesis, assisted with performing the experiments, and analyzed the data. YX is the guarantor of this work and supervised the entire project, researched the data, and wrote the manuscript.

## Supporting information


**Fig. S1.** The scatter plots of Th17 lymphocytes from kidney tissues of mice from each group are shown inside the circles. NC: non‐diabetic C57BLKS/J mice; DM: diabetic db/db mice treated with normal saline; Dap: diabetic db/db mice treated with dapagliflozin; Vog: diabetic db/db mice treated with voglibose.
**Fig. S2.** The scatter plots of Treg lymphocytes from kidney tissues of mice from each group are shown inside the circles. NC: non‐diabetic C57BLKS/J mice; DM: diabetic db/db mice treated with normal saline; Dap: diabetic db/db mice treated with dapagliflozin; Vog: diabetic db/db mice treated with voglibose.
**Fig. S3.** The scatter plots of Th17 lymphocytes from peripheral blood of mice from each group are shown inside the circles. NC: non‐diabetic C57BLKS/J mice; DM: diabetic db/db mice treated with normal saline; Dap: diabetic db/db mice treated with dapagliflozin; Vog: diabetic db/db mice treated with voglibose.
**Fig. S4.** The scatter plots of Treg lymphocytes from peripheral blood of mice from each group are shown inside the circles. NC: non‐diabetic C57BLKS/J mice; DM: diabetic db/db mice treated with normal saline; Dap: diabetic db/db mice treated with dapagliflozin; Vog: diabetic db/db mice treated with voglibose.

## Data Availability

The datasets generated and analyzed during the current study are available from the corresponding author on reasonable request.
