# Supplementary material for: Dapagliflozin reverses the imbalance of T helper 17 and T regulatory cells by inhibiting SGK1 in a mouse model of diabetic kidney disease
Source: FEBS Open Bio. 2021 May 1;11(5):1395–405. doi: 10.1002/2211-5463.13147 (PMC8091586; doi:10.1002/2211-5463.13147)
Supplement: Supplementary file 1 — Fig. S1. The scatter plots of Th17 lymphocytes from kidney tissues of mice from each group are shown inside the circles. NC: non‐diabetic C57BLKS/J mice; DM: diabetic db/db mice treated with normal saline; Dap: diabetic db/db mice treated with dapagliflozin; Vog: diabetic db/db mice treated with voglibose. Fig. S2. The scatter plots of Treg lymphocytes from kidney tissues of mice from each group are shown inside the circles. NC: non‐diabetic C57BLKS/J mice; DM: diabetic db/db mice treated with normal saline; Dap: diabetic db/db mice treated with dapagliflozin; Vog: diabetic db/db mice treated with voglibose. Fig. S3. The scatter plots of Th17 lymphocytes from peripheral blood of mice from each group are shown inside the circles. NC: non‐diabetic C57BLKS/J mice; DM: diabetic db/db mice treated with normal saline; Dap: diabetic db/db mice treated with dapagliflozin; Vog: diabetic db/db mice treated with voglibose. Fig. S4. The scatter plots of Treg lymphocytes from peripheral blood of mice from each group are shown inside the circles. NC: non‐diabetic C57BLKS/J mice; DM: diabetic db/db mice treated with normal saline; Dap: diabetic db/db mice treated with dapagliflozin; Vog: diabetic db/db mice treated with voglibose. [file FEB4-11-1395-s001.docx]

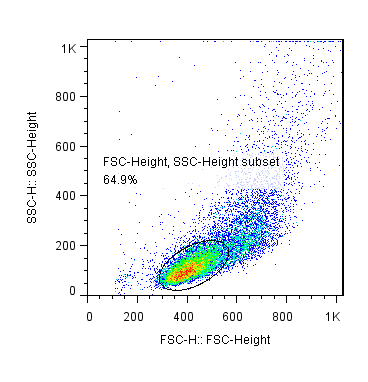

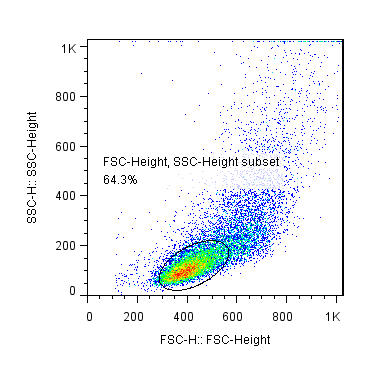

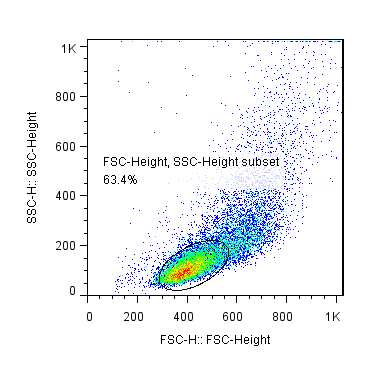

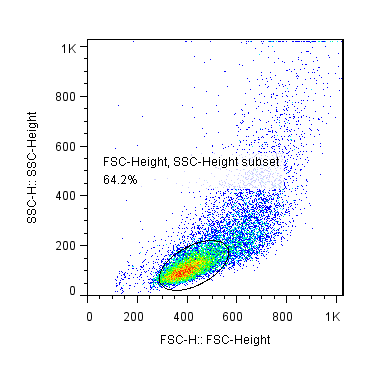


Dap

Vog

DM

NC

**Figure S1. The scatter plots of Th17 lymphocytes from kidney tissues of mice from each group are shown inside the circles.** NC: non-diabetic C57BLKS/J mice; DM: diabetic db/db mice treated with normal saline; Dap: diabetic db/db mice treated with dapagliflozin; Vog: diabetic db/db mice treated with voglibose.


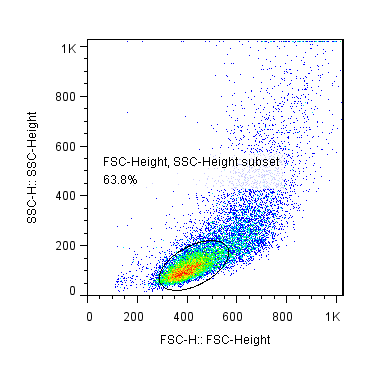

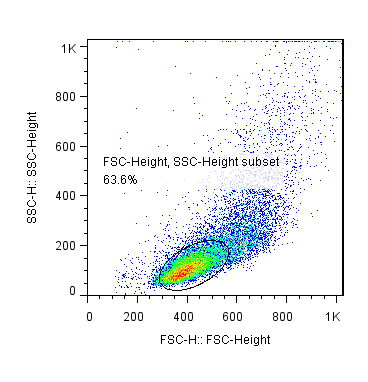


NC

DM

Vog


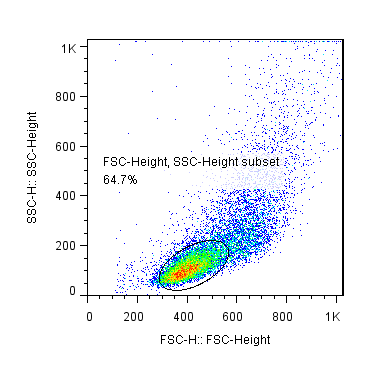

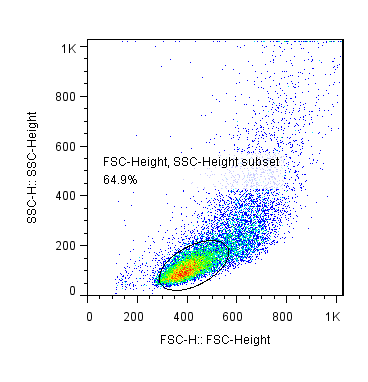


Dap

**Figure S2. The scatter plots of Treg lymphocytes from kidney tissues of mice from each group are shown inside the circles.** NC: non-diabetic C57BLKS/J mice; DM: diabetic db/db mice treated with normal saline; Dap: diabetic db/db mice treated with dapagliflozin; Vog: diabetic db/db mice treated with voglibose.


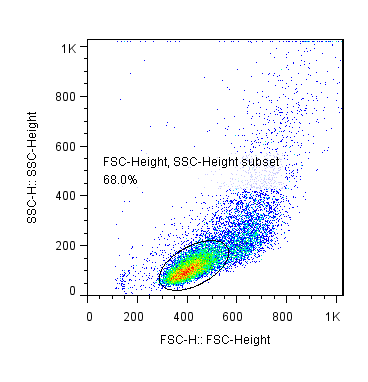

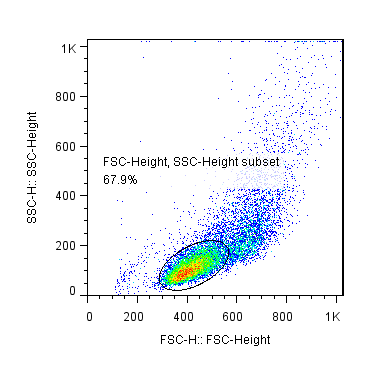

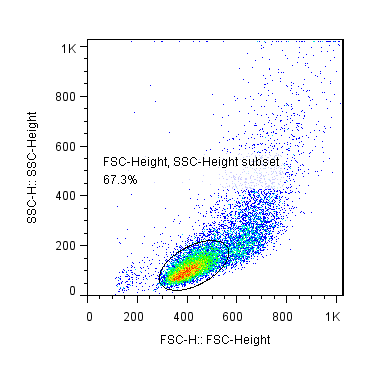

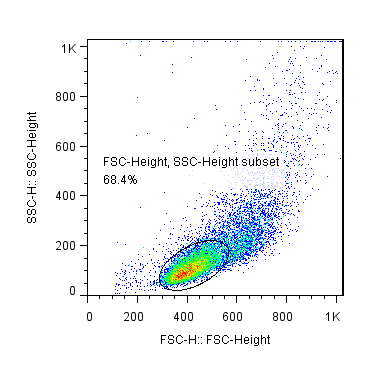


NC

DM

Vog

Dap

**Figure S3 The scatter plots of Th17 lymphocytes from peripheral blood of mice from each group are shown inside the circles.** NC: non-diabetic C57BLKS/J mice; DM: diabetic db/db mice treated with normal saline; Dap: diabetic db/db mice treated with dapagliflozin; Vog: diabetic db/db mice treated with voglibose.


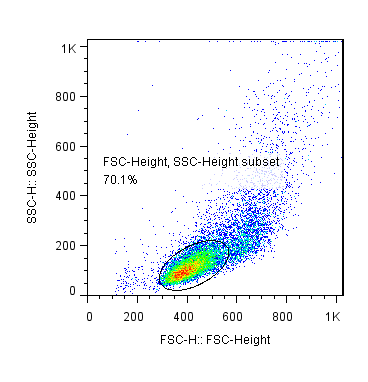

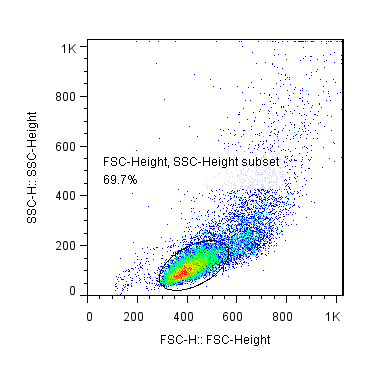


NC

DM

Dap


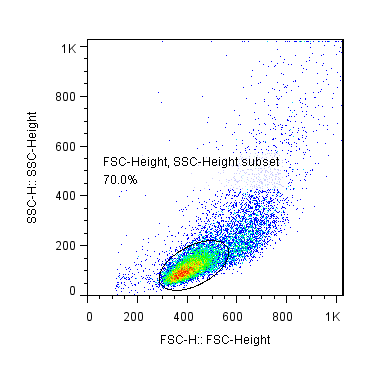

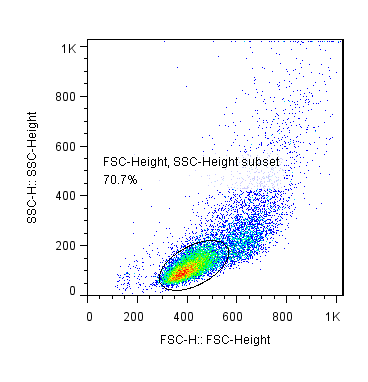


Vog

**Figure S4. The scatter plots of Treg lymphocytes from peripheral blood of mice from each group are shown inside the circles.** NC: non-diabetic C57BLKS/J mice; DM: diabetic db/db mice treated with normal saline; Dap: diabetic db/db mice treated with dapagliflozin; Vog: diabetic db/db mice treated with voglibose.
